# Gut dysbiosis and nitric oxide dysregulation in cirrhosis progression: mechanistic insights and pathophysiological implications

**DOI:** 10.1007/s13105-026-01188-w

**Published:** 2026-05-07

**Authors:** Vlad Răzniceanu, Andra Țichindeleanu, Eugen-Valentin Răducu, Șerban Ellias Trella, Iuliana Nenu

**Affiliations:** Department of Physiology, ”Iuliu Hațieganu” University of Medicine and Pharmacy, Cluj-Napoca, 400006 Romania

**Keywords:** Portal hypertension, Gut microbiota, Dysbiosis, Nitric oxide, Translational medicine

## Abstract

Cirrhosis represents the end stage of chronic liver injury, characterized by progressive fibrosis and architectural distortion that precipitate portal hypertension and systemic complications. Recent evidence positions gut microbiota dysbiosis and nitric oxide (NO) dysregulation as central, interacting pathophysiological mechanisms in cirrhosis progression. Intestinal barrier dysfunction facilitates bacterial translocation and thereby exposes the liver to lipopolysaccharides and pathogen-associated molecular patterns that trigger hepatic inflammation via Toll-like receptor signalling, a phenomenon aggravated by dysbiosis. This immune activation stimulates inducible NO synthase in Kupffer cells and systemic endothelium, generating excess NO that drives splanchnic vasodilation and worsens portal hypertension. Paradoxically, intrahepatic endothelial NO synthase activity becomes impaired, reducing sinusoidal NO availability and increasing intrahepatic vascular resistance. These interconnected disturbances perpetuate inflammation and fibrogenesis, contributing to cirrhosis decompensation and spontaneous bacterial peritonitis. Despite substantial mechanistic insight into these pathways, therapeutic translation remains limited. Statins show promise by restoring intrahepatic eNOS function and reducing portal pressure, while microbiota-targeted interventions (antibiotics, probiotics, fecal transplantation) address gut-derived inflammation. This review synthesizes our current understanding of the gut-liver-NO axis in cirrhosis, highlighting how dysbiosis and aberrant NO signalling reinforce each other through inflammatory feedback loops, and identifies critical gaps between mechanistic knowledge and clinical application that warrant further investigation.

## Key points

Dysbiosis-driven bacterial translocation sustains hepatic inflammation and fibrogenesis.

Opposing intrahepatic eNOS loss and systemic iNOS excess worsen portal hypertension.

Dysbiosis and nitric oxide overproduction form a self-reinforcing pathological loop.

Statins and microbiota therapies show promise but lack robust clinical evidence.

## Introduction

The liver is composed of two different types of cells: parenchymal cells (hepatocytes) and non-parenchymal cells: hepatic stellate cells (HSCs), liver sinusoidal endothelial cells (LSECs) and Kupffer cells (KCs) [1]. Each of these plays a part in the process that leads to cirrhosis, but the common denominator is the HSC. HSCs can be activated both directly by liver injury and by the other hepatic cells, the result of which is collagen buildup via the secretion of transforming growth factor beta (TGF-β), the most potent hepatic fibrosis inducer [2]. This leads to changes in the structure and vasculature of 0the liver, which in turn leads to hemodynamic effects and consequently to portal hypertension [3].

Nitric oxide (NO) is a molecule with a multitude of roles that depend on the target site, concentration and time of action. At very low levels it supports normal physiological functions, whereas at higher levels it can induce oxidative stress and cytotoxicity, as well as disrupt neurotransmission. It is also a potential contributor to tumorigenesis. NO is derived from L-arginine through the catalytic action of different NO synthase (NOS) enzymes: nNOS expressed in neurons, eNOS in endothelial cells and iNOS in immune cells [4]. Among the roles of NO, we find vasodilation, platelet aggregation inhibition and several contrasting effects: pro- and anti-inflammatory, pro- and antiangiogenic and induction or inhibition of apoptosis. In chronic liver disease and cirrhosis, inducible NOS (iNOS), which is up-regulated in hepatocytes, Kupffer cells and hepatic stellate cells under inflammatory conditions, produces high, sustained levels of NO that contribute to oxidative/nitrosative stress, pro-inflammatory signaling and, in many models, hepatocyte injury and fibrosis. By contrast, endothelial NOS (eNOS), particularly in liver sinusoidal endothelial cells, generates low, tonic NO that helps maintain sinusoidal homeostasis, restrains activation of hepatic stellate cells, and is a key regulator of intrahepatic vascular tone; loss of eNOS-derived NO is a hallmark of sinusoidal endothelial dysfunction in portal hypertension [5]. However, VEGF-induced eNOS activation and production of NO has also been found to cause splanchnic arteriolar vasodilation and aggravation of portal inflow in cases of PH [6].

Another stimulus of eNOS activation is bacterial translocation, common in cirrhosis and with important clinical effects [6]. The gut microbiota refers to the microorganisms (primarily bacteria) that colonise the gastrointestinal tract and have an established symbiotic relationship with their host [7]. It aids the human body in multiple ways: synthesis of vitamin K, reinforcement of the intestinal epithelial barrier via short-chain fatty acid production, and suppression of potential pathogens [8]. In cirrhosis, however, both qualitative alterations in the gut microbiota and the migration of bacterial cells and products to mesenteric lymph nodes and other extra-intestinal sites occur, thus defining bacterial translocation.

A consequence of this process is the activation of local and systemic inflammation, which in turn causes endothelial dysfunction. This translates into a series of opposing events: release of NO as a vasodilating agent, endothelin-1 as a vasoconstricting agent, as well as increased levels of asymmetric dimethylarginine, which inhibits the formation of NO. As a result, a worsening of PH and a hyperdynamic circulatory state can be noticed in patients and has been proven by a positive correlation of plasma nitrite levels with markers of hyperdynamic circulation [8].

Consequently, multiple pathophysiological disturbances involving the gut underlie and emerge from cirrhosis, interacting synergistically as the disease progresses. A focused narrative review on the interplay between NO, gut dysbiosis, and cirrhosis is warranted, given that NO overproduction plays a central role in cirrhosis-related vasodilation, portal hypertension, and renal dysfunction [9], while dysbiosis significantly amplifies this pathological NO signalling. Together, they form a triad with therapeutic relevance that remains insufficiently characterised in an integrative context. This review synthesizes evidence on the gut–liver–nitric oxide axis in cirrhosis, focusing on how intestinal dysbiosis drives hepatic inflammation and complications, how compartment-specific nitric oxide synthase activity reconciles nitric oxide’s protective and pathological roles, and how reciprocal dysbiosis–nitric oxide feedback loops sustain disease progression. This integrated framework helps explain the clinical heterogeneity of cirrhosis and may inform future therapeutic stratification.

## The gut-liver axis: liver disease, dysbiosis, bacterial translocation and inflammation

Liver cirrhosis is often accompanied by dysbiosis and intestinal barrier disruption, pointing to a link between hepatic dysfunction and alterations in the composition and activity of gut microbiota. The relationship between gut microbiota and the liver (the gut-liver axis) is bidirectional: by altering the intestinal environment (e.g., decreased motility, bile secretion), chronic liver diseases such as cirrhosis encourage dysbiosis; conversely, dysbiosis actively contributes to liver damage and the development of complications [10]. A growing body of evidence supports the idea that the severity of liver damage is closely correlated with the degree of intestinal dysbiosis in cirrhotic patients [11–13] In cirrhosis, bacterial diversity decreases, and the intestinal flora undergoes significant changes: for example, beneficial butyrate-producing bacteria (most belonging to the *Clostridium* cluster of the phylum *Firmicutes*, such as *Faecalibacterium*, *Roseburia*, *Eubacterium*, *Anaerostipes*, *Coprococcus*, *Subdoligranulum*, and *Anaerobutyricum*) [14] are severely reduced, while potentially pathogenic bacteria (Gram-negatives from the *Enterobacteriaceae* family and others) proliferate. These microbial imbalances are associated with increased levels of bacterial endotoxins in the blood and systemic inflammation, which are responsible for a suite of downstream effects that feed into the complications typically encountered in advanced cirrhosis [15]. Moreover, inflammatory responses associated with dysbiosis stimulate the expression of nitric oxide synthase, increasing nitric oxide levels and thereby exacerbating portal hypertension and vasodilation in patients with cirrhosis. Below, we detail the main documented processes by dint of which intestinal dysbiosis promotes liver cirrhosis and the ensuing fibrosis, portal hypertension, ascites, encephalopathy, infections, hepatorenal and hepatopulmonary syndromes.

### Mechanisms of dysbiosis in cirrhosis

Intestinal barrier dysfunction and bacterial translocation are important and pleiotropic actors in the pathophysiology of cirrhosis. In advanced liver disease, the integrity of the intestinal barrier is frequently impaired. By reducing blood flow to the gut, portal hypertension itself can promote ischemia and subsequent neoangiogenesis, tight junction disruption, and oedema, increasing permeability [13]. Cirrhosis can be accompanied by small intestinal bacterial overgrowth, especially in decompensated stages with portal hypertension [16] These changes in the gut allow luminal bacteria and their products to cross into the portal circulation, a process known as *bacterial translocation*. As a result, patients with cirrhosis exhibit elevated circulating endotoxin - lipopolysaccharide (LPS) levels, with portal blood LPS concentrations significantly higher than systemic levels (55.8 [42.2–79.9] vs. 23.0 [7.0–34.0] pg/ml, *p* < 0.001 as reported by R. Carnevale et al.) [17] due to the liver’s partial immune clearance [13]. Gut dysbiosis (characterised by overgrowth of Gram-negative pathobionts) is a major driver of this process, as supported by increased abundance of LPS-producing *Proteobacteria* (such as *Escherichia Coli*,* Salmonella Enteritica*,* Pseudomonas aeruginosa*), in cirrhotic microbiota, an important phylum present in the gut microbiota that is also associated with high levels of nitrites [18]. In addition to decreased clearance and heightened production of LPS, leakage resulting from impaired gut barrier functionality contributes to endotoxemia. Indeed, in vivo studies show that translocated LPS can even feedback to further weaken the intestinal barrier (via processes such as induction of enterocyte-derived NO that increases gut permeability) [19]. Altogether, a leaky gut and dysbiotic flora set the stage for persistent microbial antigen exposure to the liver.

Once bacterial products reach the liver through the portal vein, they trigger the hepatic innate immune system. Gut-derived bacterial products (especially LPS) and other pathogen-associated molecular patterns (PAMPs, such as bacterial DNA) are recognised by pattern recognition receptors such as the Toll-like receptors (TLRs) on KCs and other hepatic cells [13] LPS is a potent activator of KCs, which exhibit the highest TLR4 expression among hepatic cells, stimulating them to release a cascade of inflammatory and fibrogenic mediators [20]. This engagement triggers downstream MyD88 (Myeloid differentiation primary response 88)-dependent signalling, leading to the activation of Nuclear Factor kappa-light-chain-enhancer of activated B cells and Activator Protein-1 (NF-κB and AP-1) and subsequent rapid transcription of pro-inflammatory genes [21]. Consequently, KCs release a burst of cytokines – namely tumor necrosis factor-alpha (TNF-α), interleukins 1beta and 6 (IL-1β, IL-6) and chemokines, and generate reactive oxygen species (ROS) [22] They also upregulate iNOS, producing NO, which combines with superoxide to form peroxynitrite, amplifying oxidative stress [20].

Furthermore, this innate immune activation may be exaggerated in the gut-associated lymphoid tissue (GALT) of cirrhotic patients, with studies suggesting the production of cytokines such as TNF-α and IL-6 in response to LPS or bacterial DNA is augmented in cirrhotic patients compared to healthy individuals both at the level of the gut and systemically [9, 23]. Thus, even early-stage cirrhosis can exhibit a heightened inflammatory response to gut microbial translocation [24]. In parallel, translocated bacteria or LPS can directly activate hepatic stellate cells (HSCs) via TLR4 signalling, as HSCs in the injured liver also express these receptors [25]. Thus, activation of KCs and HSCs by microbial products links dysbiosis to both inflammation and fibrogenesis in the liver, while the hyperresponsive GALT activity exacerbates the barrier dysfunction described in the previous paragraph.

The heightened activity of immune cells at the level of the intestine and the liver has significant, cascading systemic repercussions. KCs and recruited mononuclear cells secrete large quantities of cytokines such as TNF-α, IL-1β, IL-6, and chemokines upon encountering LPS in the context of dysbiosis [13]. These cytokines contribute to positive feedback loops involved in liver inflammation and spill into the systemic circulation (contributing to a chronic low-grade inflammatory state). Clinical studies have confirmed that cirrhosis patients have significantly higher plasma levels of endotoxin and proinflammatory cytokines than healthy controls, while anti-inflammatory IL-4 and growth factor levels are correspondingly decreased [26] Notably, TNF-α levels in cirrhosis correlate directly with markers of bacterial translocation (LPS, bacterial DNA) and with the overgrowth of certain Gram-negative families (e.g. *Enterobacteriaceae*) [26]. The abundance of “harmful” gut taxa and endotoxemia shows a clear association with elevated IL-1β, IL-6, in addition to TNF-α concentrations [26]. In essence, this leads to a self-perpetuating inflammatory loop: more inflammation further damages the gut barrier and liver, allowing continued translocation. The outcome is an imbalanced cytokine milieu that favours liver injury and fibrogenesis.

Dysbiosis-related inflammation is also responsible for an uptick in the levels of the vasoactive mediator NO. Endotoxemia upregulates iNOS in multiple cell types, from Kupffer cells in the liver to endothelial cells in the splanchnic circulation [27]. Studies have shown that bacterial translocation leads to enhanced NO production; for example, cirrhotic rats with bacterial translocation have increased endothelial NOS activity and vasodilation in mesenteric vessels [28]. Clinically, patients with cirrhosis exhibit raised levels of nitrite/nitrate (stable NO metabolites) in blood, which correlate with LPS and cytokine levels [26]. The link to dysbiosis is founded on the hypothesis that overgrowth of Gram-negative LPS-producing bacteria in the gut drives NO synthesis. For instance, Efremova et al. found that *Proteobacteria* and *Erysipelatoclostridium* expansion alongside impaired gut barrier function contribute to excessive NO generation in intestinal vessels [18]. This microbiota-driven NO causes marked splanchnic vasodilation, a key factor in the hyperdynamic circulatory state of cirrhosis which ultimately supports the idea that dysbiosis-induced immune activation not only provokes inflammation but also a surge in vasodilators like nitric oxide, causally connecting altered gut microbe profiles and vascular dysfunction in cirrhosis [29].

Beyond bacterial translocation and direct immune activation, dysbiosis influences other pathways that exacerbate liver disease. One important aspect is altered microbial metabolism of nutrients and production of toxins given impaired or circumvented processing in the liver. In cirrhosis, pathobionts with urease and protease activity proliferate, leading to increased ammonia production in the gut [30]. Normally, the liver detoxifies portal ammonia via the urea cycle, but in cirrhosis, especially once portosystemic shunting occurs, this capacity is reduced, so that more ammonia reaches systemic circulation and consequently the brain. Gut dysbiosis is therefore a contributor to hyperammonemia, which has downstream effects on hepatic encephalopathy. Additionally, dysbiosis perturbs bile acid homeostasis: cirrhotic microbiomes show reduced bile acid-deconjugating and 7α-dehydroxylating bacteria, shifting the residual bile acid pool towards primary pro-inflammatory compounds [31]. This can activate inflammatory pathways (e.g. via intestinal FXR and TGR5 receptors) and further increase intestinal permeability and liver inflammation [32]. Furthermore, strong expression of FXR and TGR5 receptors in liver LSECs and macrophages were found to exert a vasodilatory effect through increased eNOS expression and inhibition of endothelin-1, showing that reduced bile acid resorption may lead to further eNOS impairment in cirrhotic patients [33]. In summary, multiple interconnected mechanisms – from microbial translocation and immune activation to metabolic dysregulation – underlie how gut dysbiosis drives cirrhosis progression.

Research in both animal models and humans highlights gut dysbiosis as a key driver of cirrhosis progression. Experimental cirrhosis models (in metabolic dysfunction-associated steatotic liver disease/metabolic dysfunction-associated steatohepatitis or alcoholic liver disease) develop an altered microbiome similar to humans, and manipulating this microbiome can impact disease outcomes [34]. For example, in one study, researchers engineered a rodent microbiota with reduced urease activity (diminishing ammonia production) and transplanted it into cirrhotic mice; the treated mice showed significantly lower ammonia levels and improved survival compared to controls [35]. Complementing this, faecal microbiota transplantation in cirrhotic rats has been shown to lower portal hypertension via a butyrate-mediated HDAC3 inhibition and PI3K/Akt/eNOS pathway in liver sinusoidal endothelial cells, directly linking microbiome composition to endothelial NO signalling and intrahepatic vascular resistance [36]. Gut-derived short-chain fatty acids (SCFAs), such as butyrate, propionate, acetate seem to inhibit the iNOS and the production of LPS-related inflammatory cytokines (TNF-α, IL-1, IL-6) by reducing NF-kB activation, though the exact mechanism is still not fully understood [37]. Furthermore, SCFAs are important local metabolites, as they promote epithelial growth and regeneration as well as the upregulation of tight junction protein expression via HDAC3 inhibition. They are also involved in the immunotolerance of intestinal macrophages through G-protein-coupled-receptors such as GPR41, GPR43, GPR109A and Olfr78 [38]. Such findings support causality between dysbiosis and host pathways that feed into the complications of cirrhosis. Since an imbalanced microbiome produces excess microbial metabolites that overwhelm the liver’s clearance capacity, the resulting endotoxemia from dysbiosis induces an exaggerated immune response, including high iNOS expression and nitric oxide release, which can disrupt vascular regulation [39] Taken together, the data positions dysbiosis as a mechanistic hub that affects ammonia handling, endothelial tone, and immune activation, pinpointing microbiota as an actionable therapeutic target in cirrhosis.

A summary of all these mechanisms is presented in Table 1.

**Table 1:** Main mechanisms by which dysbiosis contributes to cirrhosis

Mechanism	Key process	Major molecules involved	Pathophysiological implications
Bacterial overgrowth and translocation	Migration of bacteria and their metabolic products into the portal circulation	LPS, TLR4, iNOS, TNF-α, IL-1β, IL-6	Fibrogenesis in the liver via inflammation Increased splanchnic circulation via NO produced by iNOS, leading to portal hypertension
Host immunological response	Activation of Kupffer cells and hepatic stellate cells via pattern recognition receptors	LPS, TLR4, MyD88, NF-κB, AP-1, TNF-α, IL-1β, IL-6	Sustained hepatic inflammation and initiation of fibrogenesis
Vascular dysfunction	NO-mediated vasodilation in splanchnic circulation Pathological hepatic vascoconstriction by reduced activity of eNOS	NO, eNOS, iNOS, VEGF NO, eNOS, endothelin-1, ADMA	Hyperdynamic circulation, worsening portal hypertension Increased intrahepatic vascular resistance
Hyperammonemia	Increased ammonia production due to urease-producing bacteria	Ammonia	Hepatic encephalopathy, neurotoxicity
Altered bile acid metabolism	Reduced bile acid transformation by microbiota and reduced biliary acid absorption	Primary bile acids, FXR, TGR5	Increased intestinal permeability, inflammation and reduce eNOS expression in hepatic parenchyma

## Role of dysbiosis in cirrhosis progression and complications

The chronic inflammatory state triggered by dysbiosis promotes hepatic fibrogenesis. Endotoxin-activated KCs release profibrotic cytokines (like TGF-β) and mitogenic signals that stimulate hepatic stellate cells to proliferate and transdifferentiate into fibrogenic myofibroblasts, with a recent study on mice showing that untapped forms of TGF-β present in diseased livers are mobilised by activated macrophages via TNF-α and IL1β, leading to collagen production by HSCs [40]. Additionally, LPS-TLR4 signalling in HSCs lowers their activation threshold, making them more responsive to fibrogenic stimuli [25]. Over time, this persistent profibrotic state contributes to the architectural distortion (nodules and scar tissue) that defines cirrhosis. In essence, gut-derived LPS and inflammatory mediators act as a “fibrogenic switch” in the liver. There is evidence that substances such as elafibranor, a PPARα/δ (peroxisome proliferator–activated receptors) agonist which interferes with the feedback loops between LPS/TLR4 pathways and gut barrier disruption, can attenuate fibrosis progression, underscoring the causal role of dysbiosis-driven inflammation in cirrhosis [41]. We glean from these facts that dysbiosis accelerates the progression of cirrhosis by continuously activating immune cells and HSCs, leading to ongoing liver injury and scarring.

**Fig. 1:**
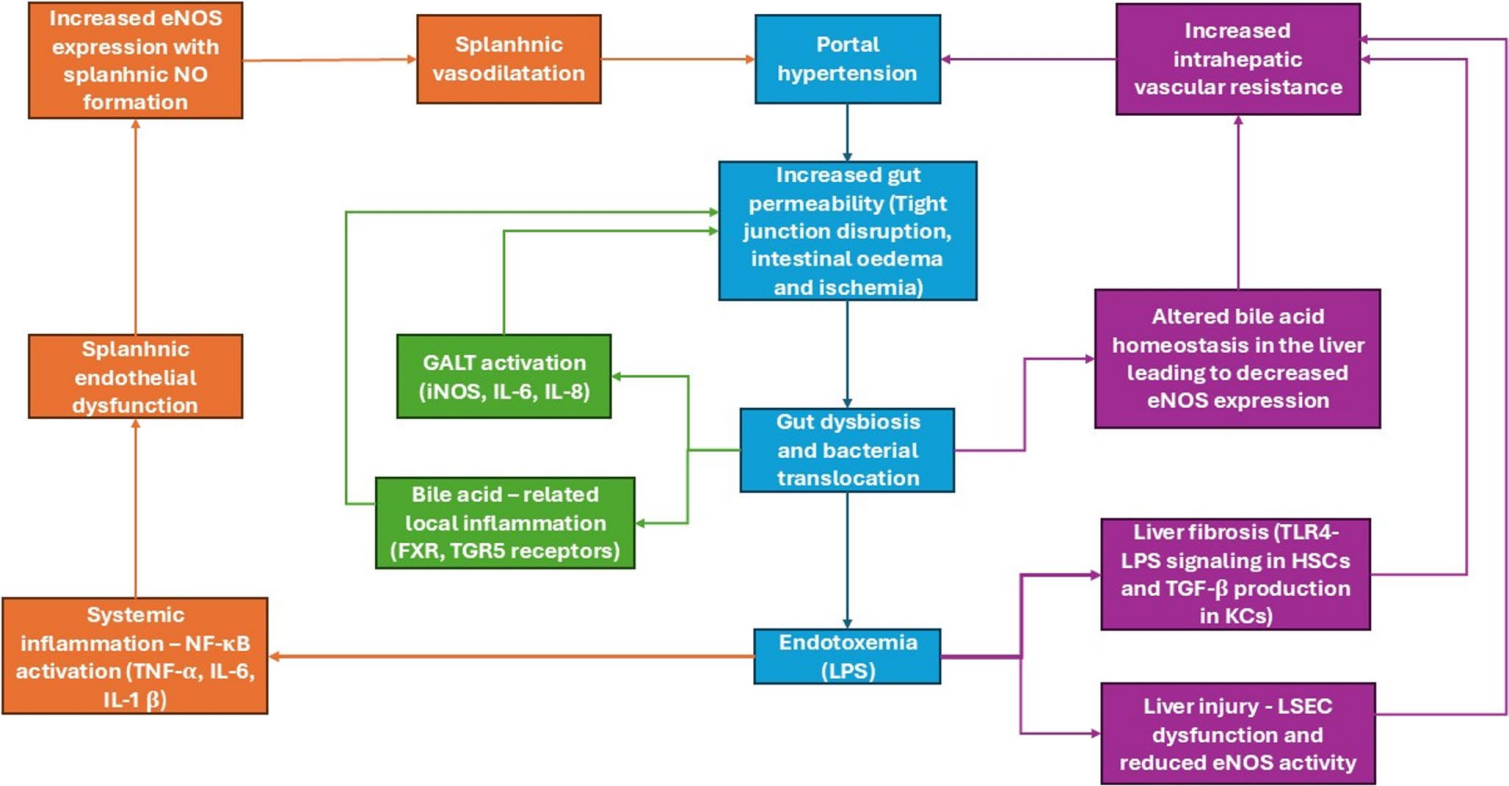
Gut dysbiosis-NO-Portal hypertension feedback loops in cirrhosis - Portal hypertension promotes increased gut permeability and intestinal ischemia leading to gut dysbiosis and bacterial translocation (blue axis). Endotoxemia leads to liver fibrosis and injury, which in turn increases intrahepatic vascular resistance worsening portal hypertension (purple loop). Systemic inflammation by LPS leads to splanchnic vasodilatation mediated by increased eNOS expression in the mesenteric vessels, augmenting portal hypertension (Orange loop). Finally, local gut inflammation in which iNOS also has an important role, aggravates the intestinal barrier directly (green loop)

Through the worsening of fibroses, intrahepatic vascular resistance rises, and portal hypertension develops. Dysbiosis plays a role in the increase in portal pressure through both structural and functional mechanisms. Structurally, increased fibrosis narrows the sinusoidal spaces; functionally, inflammatory mediators and nitric oxide cause marked vasodilation in the splanchnic circulation. Endotoxemia-induced NO lowers systemic vascular tone and triggers a hyperdynamic circulation characterised by increased cardiac output and splanchnic blood flow, which feeds into the hepatic portal system [42] It is well established that splanchnic arterial vasodilation contributes to portal hypertension and is the basis for manifestations such as ascites and hepatorenal syndrome (HRS), with recent evidence supporting the importance of an inflammatory component in their pathogenesis [43]. Bacterial translocation and the resulting endotoxemia have been linked to peripheral vasodilation and circulatory dysfunction in patients [24] with experimental animal models reporting findings that may be consistent with the NO-bacterial translocation link, namely resistance to bacterial translocation for iNOS knockout mice [44]. Overall, gut-derived inflammation promotes vasodilation, amplifying portal hypertension in a vicious cycle, where PH increases gut permeability and bacterial translocation, worsening dysbiosis and liver injury [39] to a large enough extent that a recent cross-sectional clinical study suggested the main actor in cirrhosis-specific dysbiosis may, in fact, be portal hypertension [11]. The interrelated mechanisms and positive feedback loops are presented in Fig. 1.

Portal hypertension combined with systemic vasodilation leads to one of the hallmark complications of cirrhosis: ascites. Inflammatory signals from the dysbiotic gut may worsen this by increasing vascular permeability and impairing albumin synthesis, with TNF-α and IL-6 in particular having been shown to correlate directly with ascites grade in cirrhosis [26]. As ascites progresses and systemic hemodynamics worsen, renal perfusion can decline, precipitating HRS. The hyperdynamic circulation and increased shear stress forces set off by NO and cytokines are important in HRS pathogenesis, constituting a feed-forward mechanism that enhances eNOS activity [43]. Thus, by promoting systemic and pulmonary vasodilation through NO production, leading to organ dysfunction, bacterial translocation in cirrhosis plays a pivotal role in the development of HRS. In hepatopulmonary syndrome (HPS), this process can be further evidenced by elevated exhaled NO levels and increased pulmonary expression of inducible and endothelial NOS, leading to intrapulmonary vasodilation and impaired oxygenation [45], with experimental models suggesting that endotoxemia occupies a more central role in the development of HPS relative to other factors [46].

Gut microbiota alterations are involved in the pathogenesis of hepatic encephalopathy via both hyperammonemia and endotoxemia. Cirrhotic patients with HE exhibit a significantly lower microbial diversity and a shift from health-associated commensals (e.g., *Lachnospiraceae*,* Ruminococcaceae*) toward overgrowth of urease-producing and endotoxin-rich taxa such as *Enterobacteriaceae* and *Streptococcaceae *[47] when compared to cirrhosis patients without HE. This dysbiosis increases intestinal production of ammonia from dietary protein and urea; the resulting hyperammonemia, compounded by impaired hepatic clearance, exhibits a key neurotoxic effect precipitating astrocyte swelling and neurotransmission disturbances. Notably, HE patients have reduced levels of SCFAs owing to loss of SCFA-producing microbes, which compromises intestinal epithelial tight junctions and permits greater ammonia and endotoxin absorption [48]. The translocation of microbial products activates peripheral immune cells and brain microglia, driving the release of proinflammatory cytokines that further impair blood–brain barrier integrity and neuronal function. Clinically, circulating endotoxin and inflammatory cytokine levels correlate with HE severity, supporting the concept that gut-derived inflammation exacerbates central neurological functions [49]. Importantly, the effect of interventions targeting the microbiota reinforces this pathophysiological connection: for example, non-absorbable antibiotics and faecal microbiota transplantation have been shown to reduce blood ammonia levels and improve cognition in HE, suggesting that dysbiosis is not only a marker but a driver of encephalopathy in cirrhosis [50].

Finally, perhaps the most straightforward pathogenetic course between gut dysbiosis and a complication of cirrhosis is that leading to spontaneous bacterial peritonitis (SBP), a prototypical example of bacterial translocation in advanced cirrhosis. Overgrowth of organisms like *Escherichia coli* – the most frequently identified SBP pathogen – combined with increased intestinal permeability (due to cirrhosis-related barrier dysfunction and portal hypertension) allows gut microbes and their products to migrate across the intestinal wall into mesenteric lymph nodes and ultimately the ascitic fluid, with clones of the same pathogen being found in ascitic fluid and serum [51]. Compounding this, cirrhosis-related immune dysregulation (including reduced opsonic activity in ascitic fluid and impaired neutrophil function) permits translocated bacteria to evade clearance, leading to overt infection and an intense inflammatory response in the peritoneum [52]. Dysbiosis-associated endotoxins and other microbial motifs in ascitic fluid activate peritoneal macrophages and neutrophils, unleashing a cytokine cascade that can trigger systemic inflammatory response syndrome (SIRS) and organ failures in severe cases [53]. Clinically, certain gut microbiome profiles are linked with a higher risk of SBP and related complications: cirrhotic patients with low levels of beneficial anaerobes and a bloom of *Enterobacteriaceae* have a significantly higher incidence of SBP and poorer short-term survival [54]. Conversely, therapeutic modulation of the microbiota has proven effective in preventing SBP. Selective intestinal decontamination with non-absorbable antibiotics remains standard prophylaxis, with a randomised trial demonstrating that rifaximin (a broad-spectrum antibiotic that alleviates gut dysbiosis) was superior to norfloxacin in preventing SBP recurrence, presumably by suppressing endotoxin-producing gram-negatives and improving intestinal barrier function [55]. Thus, gut dysbiosis underlies the sequence of bacterial overgrowth, translocation, and immune activation that culminates in SBP, making the intestinal microbiome a critical therapeutic target in cirrhosis.

These microbiome changes have clinical ramifications. A composite Cirrhosis Dysbiosis Ratio (CDR) has been proposed and used to quantify the shift between beneficial bacteria and potentially pathogenic taxa in the composition of the gut microbiota of patients, with stable outpatients showing no change in microbiota/CDR, patients developing hepatic encephalopathy exhibiting post-episode dysbiosis, baseline microbiota differing in a cohort of hospitalized infected cirrhotic patients MELD-matched to uninfected controls, and low CDR predicting short-term mortality and organ failure [54].

Bacterial translocation and endotoxemia stemming from dysbiosis contribute to the sequelae of portal hypertension as well. Endotoxin-triggered inflammation (via cytokines and vasoactive mediators) can elevate portal pressure and impair coagulation, thereby precipitating variceal haemorrhage [39, 56]. Consistently, cirrhotic patients with variceal bleeding show gut-barrier dysfunction and bacterial translocation, and short-course prophylactic antibiotics during bleeding episodes improve outcomes (fewer infections, rebleeding, and lower short-term mortality) [57]. Encouragingly, there is growing clinical evidence that microbiota-targeted therapies in cirrhosis can help with other complications of cirrhosis as well: probiotics (e.g., *Lactobacillus rhamnosus GG*) reducing endotoxemia and shifting taxa toward autochthonous *Firmicutes*, rifaximin producing modest compositional but measurable functional microbiome changes, and fecal microbiota transplantation improving hepatic encephalopathy in early trials, supporting the exploitation of the gut-liver axis as a therapeutic target [34].

The main cirrhosis complications and their mechanisms are presented in Table 2.

**Table 2:** Main cirrhosis complications and their underlying pathophysiology

Complication	Mechanism	Key mediators
Portal hypertension	Increased intrahepatic vascular resistance (1) (fibrosis, sinusoidal capillarization) combined with splanchnic vasodilation (2)	(1) NO, eNOS, endothelin-1, ADMA, caveolin-1, TGF-β (2) NO, iNOS, eNOS, TNF-α, IL-6, VEGF, BH4, GTP cyclohydrolase I
Ascites	Systemic and splanchnic vasodilation with inflammatory cytokine release leading to sodium and water retention	NO, iNOS, eNOS, TNF-α, IL-6, VEGF, BH4, GTP cyclohydrolase I
Hepatorenal syndrome	Renal hypoperfusion due to splanchnic blood pooling	Same as portal hypertension
Hepatopulmonary syndrome	Intrapulmonary vasodilation and impaired oxygenation due to increased pulmonary expression of iNOS and eNOS	iNOs (in pulmonary macrophages), LPS
Hepatic encephalopathy	Dysbiosis with urease-production bacteria coupled with intestinal permeability	Ammonia, LPS, TNF-α, IL-6
Spontaneous bacterial peritonitis	Bacterial translocation due to increased intestinal permeability	LPS, TNF-α, IL-6
Variceal hemorrhage	Direct effect of portal hypertension	NO, LPS, TNF-α

## Nitric oxide as mediator between cirrhosis and gut microbiota disturbances

The understanding of portal hypertension has evolved during the last decades, from the first identification of a dynamic component of hepatic vascular flow due to disruptions in vasodilatory substances, by Bathal and Grossmann [58] to the recognition by Gupta et al. of deficient NO availability as the prime culprit of portal hypertension, caused in turn by endothelial dysfunction [59]. Deriving from these, further studies demonstrated the possibility of improving endothelial dysfunction through a myriad of strategies [60] We will now summarise the current understanding of the NO function in the context of hepatic cirrhosis, followed by the aforementioned therapeutic strategies.

Physiologically, NO is synthesised by the enzyme nitric oxide synthase, or NOS. There are 4 major types of NOS, defined by their primary localisation: eNOS - endothelial NOS (found in the LSECs), iNOS - inducible NOS (expressed in macrophages, HSCs and vascular smooth muscle cells), nNOS- neuronal NOS, and mitochondrial NOS [61].

**Fig. 2:**
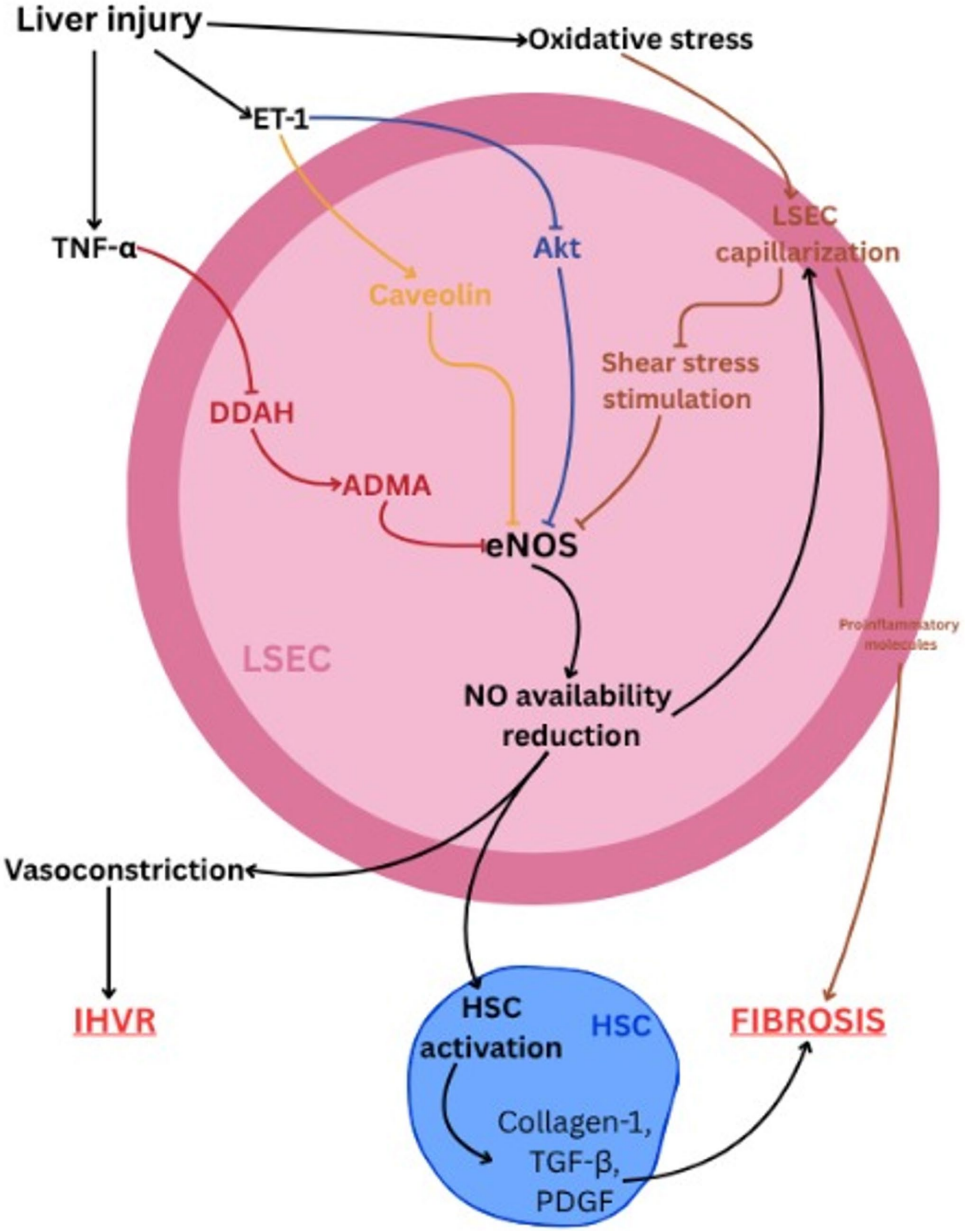
Intrahepatic NO Dysregulation in Cirrhosis - LSEC eNOS impairment driving stellate cell activation, fibrosis, and portal resistance. Liver injury and oxidative stress impair AKT-dependent eNOS activation in liver sinusoidal endothelial cells, promoting capillarization, increased intrahepatic vascular resistance, and hepatic stellate cell activation, thereby driving fibrosis

In the context of portal hypertension, eNOS is considered the primary target in NO diminution, which is a phenomenon effected to a large degree by LSECs dysfunction [60], whose causes and effects are illustrated in Fig. 2. Liver injury leads to a reduction of the activity of AKT (a serine/threonine kinase), involved in the phosphorylation and therefore induction of eNOS, through endothelin-1 stimulation [62], and the augmentation of the binding capability of Caveolin-1, a protein responsible for the inhibition of eNOS secretion [63] Also, intrahepatic oxidative stress can contribute to the impairment of the eNOS/NO axis by inhibiting AKT, as demonstrated by Gracía-Calderó et al. [64]. Furthermore, through oxidative stress and NO availability reduction, LSECs undergo a process called capillarization, which in turn leads to LSECs dysfunction; the ensuing process is poor stimulation by shear stress, but also the cells’ secretion of proinflammatory substances, which can aggravate liver fibrosis [65, 66].

Conversely, extra-hepatic vascular eNOS is overexpressed in the context of liver injury through phosphorylation in cells of the aortic area [63]; this is due to shear stress that activates AMPK [67], and to the energising of AKT, as demonstrated by Fernández-Varo et al., who successfully improved systemic haemodynamics, through the administration of an inactive AKT mutant [68]. In contrast, iNOS is expressed by resident macrophages (KCs) and hepatocytes and is induced by a variety of inflammatory cytokines [69].

**Fig. 3:**
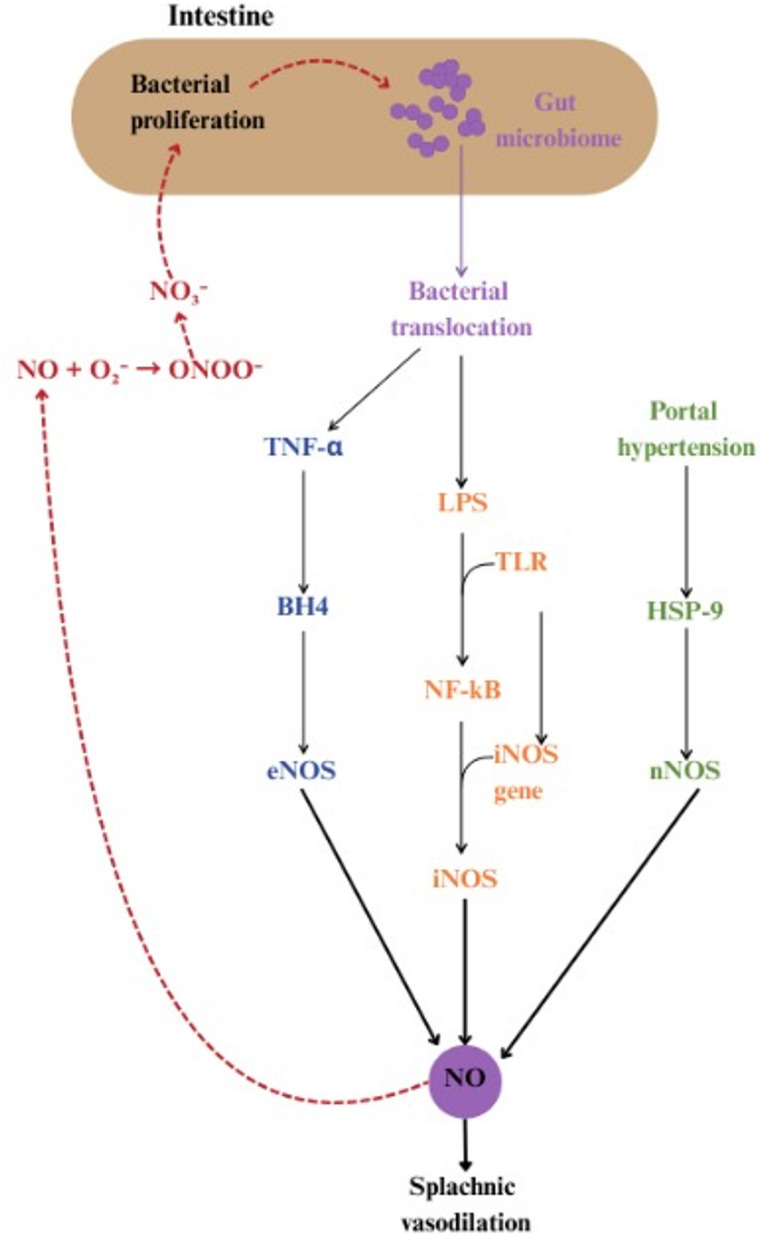
Gut-liver axis-driven Bacterial Translocation, NO overproduction and splanchnic vasodilation in cirrhosis via NO-Signaling Feedback Loop (iNOS, eNOS, nNOS Activation). Bacterial overgrowth and gut barrier dysfunction promote bacterial translocation and LPS–TLR signaling, leading to NF-κB–dependent induction of iNOS and increased NO production. Together with extrahepatic eNOS and nNOS activation, this results in increased NO bioavailability and splanchnic vasodilatation

After bacterial overgrowth and the permeabilisation of the gut barrier present in cirrhosis leads to bacterial translocation and a buildup of LPS, signalling by means of TLR determines NF-κB activation which will bind to the iNOS promoter, inducing the iNOS gene; this in turn leads to the stimulation of iNOS-derived NO production and the secretion of pro-inflammatory mediators, for instance TNF-α, which further activate the enzyme [70, 71]. These alterations lead to an increased bioavailability of NO, causing splanchnic vasodilatation as outlined in Fig. 3 [72].

Other studies also inculpate mesenteric vasculature eNOS as being a source for the inordinate amount of NO seen causing splanchnic vasodilatation in cirrhosis [63]. TNF-α seems to be one component that gives rise to NOS by expression and activation of enzymes that regulate tetrahydrobiopterin- (BH4) in mesenteric vasculature in the context of bacterial translocation [73]. Additionally, enteric nNOS appears to be stimulated by the protein-stabilising chaperone heat shock protein 9 (HSP-9), causing mesenteric vasodilatation [74]. This seems to be due to portal hypertension, as the overexpression of nNOS has been reported in portal ligation in rats and pre-hepatic portal hypertension [75, 76]. Additionally, persistent inhibition of nNOS noticeably alleviates systemic hyperdynamic circulation [77].

We will now consider the effects NO has on gut microbiota. Bacteria have an established role in the production and intricate metabolism of nitrogen, including NO and can be an origin of NO and its inflammatory derivatives [78]. Albeit inflammation often damages microorganisms via its array of metabolites, certain commensal bacteria have been noted to profit from this by metabolizing certain radicals and enlarging their population leading to dysbiosis. This has been observed in the increase of *Enterobacteriaceae* populations in the context of inflammation, particularly *E. coli* and *S. typhimurium* [79]. One antimicrobial mechanism of host inflammatory cells is the production of nitric oxide (NO), which can react with superoxide (O₂·⁻) to form peroxynitrite (ONOO⁻), a reactive species that can subsequently decompose to nitrate (NO₃⁻); this can be employed as a terminal electron acceptor in anaerobic respiration via denitrification, particularly in *Gammaproteobacteria* [80]. NO produced by iNOS has been viewed as an important antimicrobial agent produced by the host, directly or through the induction of antimicrobial peptide expression [79, 81]. Despite this, NO can augment the population expansion of denitrifying facultative anaerobic bacteria, like *E. coli*, *S. typhimurium* and *Gammaproteobacteria* [80]. Furthermore, a positive feedback loop has been proposed between intestinal bacteria and inflammatory cells: inflammatory damage and increased gut permeability lead to enhanced release and translocation of LPS from Gram-negative bacteria, thereby amplifying the inflammatory response [82]. Alterations in gut microbiota is present in patients with cirrhosis, and MELD scores have been positively corelated with the proportion of gut *Enterobacteriaceae* [54] It can be presumed that NO produced in the context of liver cirrhosis can be implicated in similar interactions with the microbiota.

## Role of eNOS in cirrhosis

It is widely accepted that in cirrhosis, while there is a normal expression of eNOS in the liver parenchyma, there is a significant reduction in intrahepatic eNOS activity via post-translational modifications and the presence of eNOS inhibitors [83] Dysregulation of eNOS in LSECs via abnormal Akt signaling, deficiency of BH4 [84], or by eNOS binding to caveolin-1, blocking the calmodulin-mediated activation of eNOS [85] leads to decreased NO availability and thus, a vasoconstriction of the sinusoid vessels and an increase in intrahepatic vascular resistance (IHVR) [84] LSECs present fenestrae without any basal membrane, making them unique in mammalian organisms. Maintenance of these characteristics via the VEGF-Akt-eNOS axis is essential for HSC quiescence [71] A reduction in eNOS activity translates to LSEC capillarisation (secretion of basal membrane collagen type IV and laminin along with loss of the fenestrae), which in turn leads to activation of HSC, which secrete extracellular matrix, collagen type 1, and pro-fibrotic mediators such as TGF-β and PDGF, leading to fibrosis [85]. Activated HSCs alter liver architecture by secreting extracellular matrix proteins; their contractility is enhanced due to expression of α-SMA (α- smooth muscle actin), leading to increased pressure via contraction mediated by endothelin-1, angiotensin 2, and norepinephrine [86]; they also further promote LSEC capillarisation, increasing IHVR [87]. Hepatocytes, along with NO-donor supplementation, cannot maintain HSCs’ quiescence and thus indicate a paracrine role of normal LSEC in HSC quiescence [71]. Reducing NO availability also facilitates thrombosis formation [87].

Additionally, increased oxidative stress and exposure to TNF-α impair the metabolism of eNOS inhibitor asymmetric dimethylarginine (ADMA) by downregulating dimethylarginine dimethylaminohydrolase (DDAH), the enzyme responsible for the breakdown of ADMA [83].

Soluble guanylyl cyclase is the intracellular target of NO that catalyzes the production of cGMP, the mediator responsible for vasodilation [88]. In hepatocytes, there is a difference in expression of the main phosphodiesterase that breaks down cGMP, phosphodiesterase 5 (PDE5), depending on the zone of hepatic parenchyma. PDE5 is poorly expressed in perisinusoidal zones, and there is a sharp increase in expression in zone 3 of the hepatic lobules. This zonation is perturbed in the development of cirrhosis, further increasing IHVR and endothelial dysregulation [89].

Increased splanchnic arterial flow occurs in PH due to increased circulating vasodilators, decreased vascular response to vasoconstrictors, and autonomic dysfunction. eNOS expression and activity are enhanced in mesenteric and portal circulation by shear stress, upregulation of GTP cyclohydrolase I (essential in biosynthesis of BH4 co-factor of eNOS) mediated by bacterial translocation, coupling of eNOS with heatshock protein 90, and phosphorylation (and thus activation) of eNOS by Akt [84, 90]. Regarding the Akt pathway activation, VEGF is also increased in the mesenteric microvasculature in cirrhotic and PH mice and induces eNOS endothelial expression [91]. Its secretion can be stimulated by hypoxia in the context of liver fibrosis, and it binds to VEGFR on the endothelium, leading to Akt phosphorylation and thus contributes to vasodilation and angiogenesis in cirrhosis [90].

## Role of iNOS in cirrhosis

LPS represent an important trigger for iNOS induction, especially in immune cells. An early source of inflammation in cirrhotic patients might be the activated CD14 + duodenal macrophages that express iNOS and produce NO, IL-6, and IL-8, which lead to an increased permeability of the intestinal barrier and an elevation of serum LPS, generalising the inflammation and induction of NO [92]. Kupffer cells activated by LPS and inflammatory cytokines lead to the secretion of TNF-α, TGF-β, and IL-6, which in turn activate HSCs [93].

**Fig. 4:**
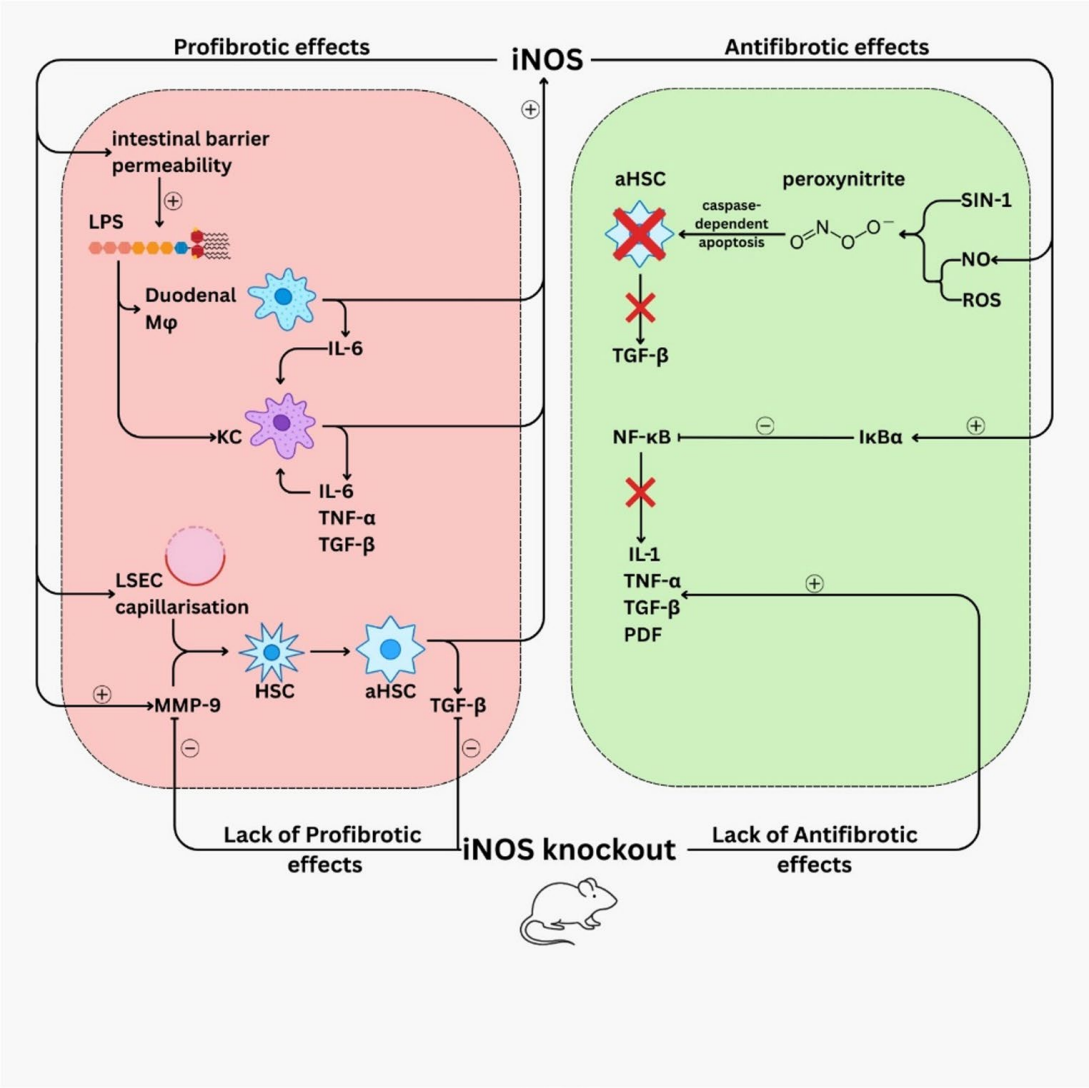
Evidence of dual profibrotic and antifibrotic roles of inducible nitric oxide synthase (iNOS) in chronic liver disease. iNOS promotes profibrotic pathways via increased intestinal barrier permeability, macrophage activation, LSEC capillarisation and increased MMP-9 expression, while also exerting antifibrotic effects through peroxynitrite formation, inhibition of inflammatory signaling, and apoptosis of activated HSCs. iNOS knockout abolishes both effects

There is conflicting evidence in the literature on whether the increased iNOS expression and activity alleviate or further exacerbate liver fibrosis, the data underlying these contrasting mechanisms being summarized in Fig. 4.

On the one hand, iNOS can facilitate LSEC capillarisation (i.e., losing their fenestrae and the ability to maintain HSCs quiescence), and can increase MMP-9 expression, a key factor in HSCs activation [93].

In the context of systemic inflammation with high levels of reactive oxygen species (ROS), iNOS induction in hepatocytes, KCs, and resident monocytes and macrophages will increase the formation of reactive nitrogen species such as peroxynitrite. A study on the role of iNOS in mice fed with a high cholesterol diet for 6 weeks showed that iNOS knockout mice presented similar levels of hepatic injuries (similar serum ALAT and ASAT levels) as wild-type mice, but significantly lower fibrosis mediators, such as TGF-β, TNF-α, and MMP-9 stain on liver histology, pointing to a role of iNOS in fibrogenesis [94]. Consistent with these findings is also the work of Jinhang Zhang et al. In their model, using SKLB023, an iNOS inhibitor on metabolic induced fibrosis led to an overall improvement of the fibrosis and steatosis by inhibiting the TGF-β/Smad pathway [95]. In vivo studies with mice with CCl₄-induced cirrhosis, iNOS knockout mice showed significantly decreased fibrosis, but more hepatic apoptosis [96].

Contrastingly, another study with mice fed a high-fat diet for 48 weeks showed the importance of iNOS in inhibiting NF-κB via stabilisation of inhibitory kappa B alpha factor (IκBα) and thus in reducing inflammation and fibrosis by inhibiting NF-κB dependent secretion of IL-1, TNF-α, PDGF, and TGF-β, pointing toward the protective effects of long-term high dose of NO. Furthermore, using NO and superoxide anion donor SIN-1 in cultures of rat liver HSCs to mimic the microenvironment in chronic liver disease, it was found that peroxynitrites can induce HSC apoptosis in a caspase-dependent manner, which can alleviate fibrosis [97]. In a long-term high-fat diet model, absence of inducible nitric oxide synthase was associated with increased hepatic inflammation and fibrosis despite marked reduction in steatosis, indicating that iNOS may exert distinct, context-dependent fibrogenic effects depending on the underlying pathophysiology [98, 99].

Adiponectin, an adipocytokine with antiinflammatory and antifibrotic properties, was shown to activate the iNOS/NO pathway in HSC via adipoR2 receptors following the adipoR2-AMPK-JNK/Erk1/2-NF-κB axis, inhibiting HSC proliferation and promoting HSC apoptosis while also stimulating the eNOS/NO pathway in LSECs [100].

It seems that hepatocyte-derived iNOS has a protective role in NASH pathogenesis through HO-1 upregulation via augmented Nrf-2 nuclear transcription. The iNOS KO mice developed extensive steatosis and liver injury compared to wild type mice [101]. It is also worth noting that there are different potential patterns of iNOS expression in liver disease in relation to the pathogenetic mechanism. Interestingly, it has been demonstrated that in bile-duct-ligated models of cirrhotic rats the iNOS expression was highest during active fibrosis compared to metabolic or toxic induced cirrhosis models [102]. Other findings indicated iNOS expression is upregulated in bile-duct-ligated models until week 3 but afterwards it is reduced, suggesting that hepatocytic iNOS has a more acute implication in fibrosis rather than long-term [103].

Overall, these findings highlight the dual action of iNOS in chronic liver disease: on one hand promoting fibrogenic pathways via LSEC capillarisation and HSC activation in toxic or bile-duct-ligated models, while, on the other hand, in metabolic-induced NASH models, it seems to have a bigger role in prevention of lipid accumulation and an antiinflammatory role via NF-κB suppresion and an induction of HSC apoptosis.

The overactivation of iNOS and eNOS leads to a potent vasodilatation, which is responsible for effective arterial hypotension and the activation of SRAA that aggravates the retention of water and sodium. Effective hypovolemia also induces HIF-1 alpha expression and leads to increased VEGF [87]. PH leads to increased bacterial translocation, further contributing to the vasodilation [86]. Table 3 summarizes the distinct roles of nitric oxide across physiological compartments, detailing the sources of nitric oxide synthase, mechanisms of dysregulation, and their respective pathophysiological consequences.

**Table 3:** Compartment-specific alterations of nitric oxide synthase in cirrhosis and their pathophysiological consequences

Location	NOS type	Alteration in cirrhosis	Mechanism	Pathophysiological Effects
Liver sinusoidal endothelial cells	eNOS	Decreased activity	Inhibition of AKT, caveolin-1 binding, deficiency of BH4	Vasoconstriction and increased intrahepatic resistance. Fibrosis by HSC activation
Kupffer cells	iNOS	Upregulated	TLR4–NF-κB activation by LPS	Increased oxidative stress and pro-inflammatory signaling leading to hepatic injury and fibrosis
Splanhnic circulation	eNOS	Overactivation	Shear stress, AKT activation, upregulation of GTP cyclohydrolase I	Portal hypertension, ascites, increased intestinal permeability
Splanhnic circulation	iNOS	Overactivation	Cytokines from LPS stimulation (TNF- α, IL-6, IL-1β)	Portal hypertension, ascites, increased intestinal permeability
Intestinal immune cells	iNOS	Upregulated	LPS stimulation	Increased intestinal permeability, systemic inflammation
Enteric nervous system	nNOS	Upregulated	HSP-90 activation, portal hypertension	Mesenteric vasodilation

### Therapeutic implications

Treatment in cirrhosis focuses on easing the patients’ symptoms and targets the various complications emerging from this state [104]. There are a few options which target NO and its effects. Curcumin seems to have anti-inflammatory and antioxidant effects, and it has been found to reduce the expression of eNOS, thus limiting the formation of NO. It also improves LPS clearance in the liver, thus lowering the effects of bacterial translocation on the liver. This mechanism is mediated by another compound, kahweol, which naturally occurs in coffee beans [104].

In the context of cirrhosis, there is also strong evidence that statins improve intrahepatic endothelial function by improving eNOS activity and reducing pathological vasoconstriction. Mechanistically, statins inhibit 3-hydroxy-3-methylglutaryl-CoA reductase, preventing the formation of isoprenoids required for RhoA GTP-ase activation. This leads to reduced RhoA/Rho-kinase (ROCK) signalling, which otherwise suppresses eNOS activity by inhibiting eNOS and promoting hepatic stellate cell (HSC) contraction [105]. Concurrently, statins rapidly induce phosphorylation and subsequent activation of the PI3K-Akt pathway in endothelial cells, resulting in Akt-dependent phosphorylation of eNOS at Ser-1177 and increased NO synthesis [106, 107]. Collectively, these actions increase hepatic NO bioavailability and promote intrahepatic vasodilation. Preclinical studies have demonstrated these effects: simvastatin or atorvastatin treatment in cirrhotic rats significantly upregulates eNOS mRNA and protein, enhances eNOS phosphorylation and cGMP production, and lowers Rho-kinase activity in the liver [105]. These molecular changes translate into hemodynamic benefits, as statin-treated cirrhotic rats show a reduction in intrahepatic vascular resistance and portal pressure without systemic hemodynamic changes. In carbon tetrachloride-induced cirrhotic rats, short-term simvastatin increased hepatic eNOS expression and Akt-mediated eNOS activation, resulting in higher liver NO levels and improved endothelial vasodilator responses [108]. Clinically, these mechanisms confer therapeutic benefits in portal hypertension, reducing liver fibrosis progression and risk of variceal bleeding, as highlighted by a 2017 meta-analysis [109]. An acute study in patients with cirrhosis showed that a single 40 mg dose of simvastatin increased hepatosplanchnic NO metabolite release and significantly decreased hepatic sinusoidal resistance, thereby improving portal flow without affecting systemic pressure [107]. In a randomised controlled trial, one month of simvastatin therapy reduced the hepatic venous pressure gradient (HVPG) by ~ 8% in patients with portal hypertension, with greater HVPG declines (11%) when simvastatin was combined with non-selective β-blockers [110]. Importantly, simvastatin also improved hepatic perfusion (indocyanine green clearance), suggesting enhanced effective microcirculatory flow, and it had no significant safety issues in these patients [110].

Beyond their hemodynamic effects, statins exert anti-inflammatory and anti-fibrotic actions in cirrhosis. Statins reduce endothelial inflammation by inhibiting NF-κB activation and down-regulating adhesion molecules (e.g. ICAM-1/LFA-1), which leads to lower release of pro-inflammatory cytokines such as TNF-α and IL-6 [104]. In cirrhotic rat models, statins have been shown to blunt inflammatory cascades triggered by bacterial lipopolysaccharide (LPS): in a sepsis-induced acute-on-chronic liver failure model, simvastatin administration prevented most LPS-driven complications and normalised the surges in TNF-α and oxidative stress, markedly improving survival [111]. Statins also directly target HSCs, the key fibrogenic cells in cirrhosis. By inhibiting the RhoA/Rho-kinase pathway, statins block the post-translational geranylgeranylation of RhoA that enables its translocation to the cell membrane, a critical step for its activation. As a result, RhoA remains in its inactive cytosolic form, preventing downstream activation of Rho-kinase and the subsequent contractile and fibrogenic responses, reducing HSC myosin light chain phosphorylation and contractility and thus limiting sinusoidal constriction [112]. Collectively, these pleiotropic effects - promoting NO-mediated vasodilation while dampening inflammation and fibrosis - underline the therapeutic effectiveness of statins in cirrhosis. By enhancing eNOS activity and NO production in the intrahepatic circulation (through Akt-dependent eNOS phosphorylation and RhoA/ROCK inhibition) and by modulating immune-fibrogenic pathways, statins can improve portal hypertension and slow cirrhotic progression.

Knowing that one of the pieces in the puzzle of cirrhosis pathophysiology is increased levels of ROS produced by mitochondria, an additional avenue has been explored in the literature – a mitochondria-targeted antioxidant, mitoquinone. This was found to reduce the expression of iNOS and portal pressure in rat models of cirrhosis [104]. Another antioxidant and frequently used therapeutic agent in cirrhosis is silymarin. Both used on their own, but also in combination with taurine, it was found to lead to significantly reduced NOS activity and NO levels in rats with induced cirrhosis. On the other hand, the combination with taurine was also shown to cause an increased activity of superoxide dismutase, an undesirable result, indicating that further optimization of such combination treatment protocols is required [104].

In the quest to combat bacterial translocation, two main approaches are generally considered: trying to restore and improve the body’s natural defences by using either probiotics or faecal microbiota transplantation, or eliminating the etiopathogenetic vector entirely, by selective bacterial decontamination. In the first case, probiotics can not only correct gut dysbiosis [113], but also increase the production of short-chain fatty acids [114] and reduce intestinal permeability [115]. One choice of probiotic is VSL#3, which was found to increase NO blood levels after 28 days of use [116]. Even if they do not act directly on the production of NO, probiotics influence its levels by reducing LPS and bacterial translocation [117]. Regarding faecal microbiota transplantation, a clinical study aiming to establish the safety of this therapeutic method for patients with hepatic encephalopathy found that it not only is safe, but it also leads to improved duodenal mucosal diversity, dysbiosis and reduced LPS-binding protein expression [118]. In the second case, a study with 102 cirrhotic patients and 30 controls found that using norfloxacin for 4 weeks led to a reduction of serum levels of LPS-binding protein as well as nitric oxide metabolites [119]. The issue, however, with using antibiotics is the emergence of resistant bacteria. This means other options must be taken into consideration, one of which is the use of propranolol, which can reduce the amount of bacteria in the gut by accelerating intestinal transit [120]. The advantage of using propranolol is that it is already being used as a prophylactic measure against variceal bleeding, as it lowers PH, which further helps the issue as it leads to lower rates of bacterial translocation [121]. 

While we have not found trials in the literature that focus both on nitric oxide and bacterial translocation, there are some that explore NO modulation in cirrhosis. One of them discovered the beneficial effects of a new NO-releasing agent on cirrhotic rats: it prevented ascites formation, reduced portal pressure and liver collagen levels, thus lowering intrahepatic resistance without impacting systemic circulation [122]. Another trial used liver-targeted nanoparticles to deliver NO in cirrhotic rats which resulted in a marked decrease of aspartate aminotransferase, lactate dehydrogenase and PH compared to the controls. The systemic circulation parameters were not altered in this instance either, while collagen fibre quantities and activation of HSC were lower than in the control group, proving the efficiency of these nanoparticles [123].

A summary of the therapeutic agents, their primary targets, clinical effects and limitations is presented in Table 4.

**Table 4:** Current and potential therapeutic agents in cirrhosis: primary target, clinical effects, limitations and strength of evidence for clinical use

Therapeutic agent	Primary target	Clinical effects	Strength of evidence	Limitations
Curcumin	Inhibition of eNOS in systemic circulation Increased hepatic LPS clearance	Ameliorates splanchnic hyperdynamic circulation Reduces LPS-related inflammation	E - experimental	Specific anti-inflammatory effects have not been addressed in humans
Statins	Increased liver eNOS expression by Akt-dependent phosphorylation and inhibition of RhoA/Rho-kinase pathway Inhibition of NF-κB activation	Reduces intrahepatic vascular resistance (improved endothelial function and suppressed HSC contraction) which in turn reduces portal hypertension without affecting systemic pressure Dampening of LPS-induced inflammation	A – Meta-analyses and randomized clinical trials	Limited data in patients with advanced cirrhosis
Non-selective β-blockers	Decrease in portocollateral blood flow Splanchnic vasoconstriction Increased gut mobility, avoiding bacterial overgrowth	Reduces the hepatic venous pressure gradient (HVPG) with systemic pressure effects. Reduces bacterial translocation and esophageal varices bleeding	A – standard of care in portal hypertension	15% of patients may have contraindications and an additional 15% may not tolerate β-blockers [124]
Silymarin	Reduction in iNOS activity Reduced levels of reactive oxygen species	Possible improvement of biochemical liver parameters and Child-Pugh score	D – limited, heterogeneous clinical evidence	Mixed results
Probiotics	Reducing LPS and bacterial translocation	Reversal of minimal hepatic encephalopathy	B – meta-analysis of well-designed cohorts	Minimal use
Fecal microbiota transplantation	Improved duodenal mucosal diversity Reduced LPS-binding protein expression	Reduces the severity of hepatic encephalopathy	B – limited large-scale trials	Lack of sufficient research on large patient groups to formulate a new treatment protocol [124]
Antibiotics	Lowers the serum levels of LPS-binding protein by reducing pathogenic bacteria	Reduced blood ammonia improving hepatic encephalopathy Prevents spontaneous bacterial peritonitis	A – standard of care in hepatic encephalopathy alongside lactulose	Emergence of resistant bacteria
NO-Releasing Agents/Nanoparticles	Direct NO delivery to the liver	Marked decrease in aspartate aminotransferase, lactate dehydrogenase and portal hypertension	E - experimental	Limited clinical data

Despite significant mechanistic insights into nitric oxide (NO) dysregulation in cirrhosis, effective targeted therapies have yet to emerge in clinical practice. As the evidence indicates, endothelial dysfunction and aberrant NO production play important roles: endothelial NO synthase (eNOS) activity is impaired in cirrhotic livers, while bacterial translocation in cirrhosis triggers inflammatory cascades (e.g. TNF-α elevation) that enhance eNOS-derived NO release and vasodilation. Interventions that have been explored accordingly, for example statins, may restore endothelial NO by up-regulating eNOS, while antioxidants aim to improve NO bioavailability (e.g. by preserving eNOS cofactors and limiting oxidative NO scavenging), and microbiota-targeted therapies (such as non-absorbable antibiotics or probiotics) seek to reduce gut-derived inflammatory signals that exacerbate NO imbalance.

However, translating these strategies into durable treatments has been challenging. There is still progress to be made for the establishment of a comprehensive arsenal in the management of cirrhosis, a notable example being that no specific pharmacotherapy for severe complications like acute-on-chronic liver failure is approved to date beyond supportive care [125]. Furthermore, even the use of pharmacotherapies such as statins, whose promise in reducing portal pressure is well-documented, still rests on evidence gathered mainly from studies where most patients did not have advanced cirrhosis, and safety and long-term efficacy remain uncertain: as patients with decompensated cirrhosis are typically excluded from large trials, the true risk of drug-related toxicity (e.g. statin-induced liver injury or myopathy) in this population remains unknown [126].

A further reason for this translational gap is that, as previously shown, NO dysregulation in cirrhosis is heterogeneous, requiring actionable compartment-specific and stage-dependent interventions instead of indiscriminate regulation of nodes in its pathophysiology. NO deficiency increases sinusoidal tone and portal resistance, whereas excess splanchnic and systemic NO contributes to vasodilation, effective hypovolaemia, and circulatory dysfunction [127]. Thus, therapies that globally enhance NO signalling may improve one vascular bed while worsening another, which helps explain why mechanistically attractive interventions have repeatedly underperformed in human studies. More broadly, interventions that improve surrogate haemodynamic or biomarker endpoints may not translate into lower rates of decompensation, acute-on-chronic liver failure, or death; accordingly, the phase III LIVERHOPE trial found no such benefit with a simvastatin-rifaximin combination in decompensated cirrhosis, suggesting that correction of selected mechanistic pathways may be insufficient in advanced disease [128].

For example, the liver-targeted NO donor NCX-1000 and the tetrahydrobiopterin analogue sapropterin were both safe in patients with cirrhosis but failed to significantly reduce portal pressure, indicating that correction of a single molecular defect is often insufficient in advanced human disease [129, 130]. A similar limitation applies to microbiota-directed strategies: although biologically plausible, they are difficult to standardise, and in the recent THEMATIC trial the apparent benefit of faecal microbiota transplantation depended in part on donor engraftment and baseline microbial composition, underscoring the importance of host-specific response [131]. This may provide grounds for cautious optimism, as emerging clinical and multi-omic data suggest that microbiota-targeted interventions may become more effective when guided by host-specific factors, opening the way to more precisely tailored and biologically informed therapies.

These limitations help answer the question of why, with such detailed mechanistic knowledge on the role of NO and gut microbiota in the pathophysiology of cirrhosis, more effective treatments have not expeditiously emerged. A more foundational explanation in response to this question is the highly complex nature of cirrhosis: it involves a “complex network of many interacting and redundant pathophysiological pathways” [132], so that targeting a single node (such as NO or the gut microbiota) may be insufficient due to the intricate compensatory mechanisms involved. Altogether, this gap between strong mechanistic insight and the absence of effective clinical therapies underscores the need for integrative, multi-targeted strategies supported by well-designed translational studies. While enhanced eNOS activity is generally considered beneficial, its modulation should be investigated further in combination with other therapeutic approaches to clarify its complex clinical impact.

## Conclusion

As an organ with an exceptionally broad functional repertoire, the liver maintains systemic homeostasis through finely balanced processes. Consequently, hepatic injury produces widespread downstream effects, as exemplified in cirrhosis and further amplified by the complex, bidirectional interplay between liver damage and the intestinal milieu. Within this framework, nitric oxide emerges as a pivotal mediator with dual, context-dependent roles: dysregulated nitric oxide production contributes to intrahepatic endothelial dysfunction and pathological vasodilation, while physiologic nitric oxide signaling – particularly eNOS-derived nitric oxide – exerts protective effects by preserving sinusoidal homeostasis, vascular responsiveness, and anti-inflammatory balance. This review underscores the importance of therapeutic strategies for advanced liver disease that integrate current insights into gut microbiota-liver interactions and nitric oxide signalling, recognizing both the detrimental and protective dimensions of nitric oxide in disease initiation and progression.

## Data Availability

No datasets were generated or analysed during the current study.
